# Epigenetic Reprogramming by *Mycobacterium tuberculosis* Secretory Proteins: Implications for Pathogenesis and Therapy

**DOI:** 10.3390/antibiotics15060557

**Published:** 2026-05-30

**Authors:** Krishna RV, Nafsiya Asif, Akash N. Sethunath, Deepak T. Thekkumkara, Devanandana Binu, Gowri Krishna, Aarsha A. Sureshkumar, Arjun M. Menon, Shwetha Susan Thomas, Kuniyil Abhinand, Abhinav Sasikumar, Sandhya Padmakumar, Ardhra Paniker, Pradeesh Babu, Geetha B. Kumar, Bipin G. Nair, Aravind Madhavan

**Affiliations:** School of Biotechnology, Amrita Vishwa Vidyapeetham, Amritapuri, Kollam 690525, Kerala, India

**Keywords:** tuberculosis, epigenetic reprogramming, nucleomodulins, DNA methylation, histone modification, chromatin remodeling, host directed therapy, antimicrobial resistance

## Abstract

*Mycobacterium tuberculosis* (Mtb) continues to pose a significant global health risk, primarily due to its capacity to modulate host immune responses and achieve prolonged persistence. Recent evidence has increasingly underscored the significance of epigenetic reprogramming as a principal mechanism through which Mtb modifies host cellular functions without altering the fundamental DNA sequence. This review gives a full picture of how Mtb secretory proteins work as nucleomodulins to directly target host chromatin and control gene expression. Mtb uses special secretion systems, such as the ESX (Type VII) and SecA2 pathways, to enable effector proteins to enter host cells. Some of these proteins move to the nucleus and interact with machinery that is linked to chromatin. These nucleomodulins facilitate various epigenetic modifications, encompassing non-canonical histone methylation, DNA methylation, and the modulation of histone acetylation, resulting in extensive transcriptional reprogramming of immune-related genes. These changes make important host defence mechanisms less effective, such as macrophage activation, antigen presentation, cytokine production, and antimicrobial responses. This helps bacteria survive and avoid the immune system. Epigenetic remodeling also affects the polarization and metabolic states of macrophages, which further affect the progression of disease. The reversible characteristics of epigenetic modifications offer a significant prospect for host-targeted therapeutic strategies. Targeting enzymes such as histone deacetylases and DNA methyltransferases has shown potential in restoring immune function and enhancing bacterial clearance, particularly when used in combination with conventional anti-tubercular therapies. Even with these improvements, there are still big problems with fully understanding the functional diversity of Mtb secretory proteins and turning these discoveries into useful medical tools. In general, understanding how Mtb-secreted nucleomodulins and host epigenetic regulation interact is important for understanding how tuberculosis works and finding new ways to treat it.

## 1. Introduction

Tuberculosis, caused by Mtb, is a highly transmissible infectious disease which is spread through the aerosols released by active TB patients. It remains the leading cause of death worldwide and is ranked among the top 10 causes of global death. Although the disease typically targets the lungs (pulmonary tuberculosis), it can also spread to other parts of the body (extrapulmonary Tuberculosis) [1]. Mtb may persist in a dormant state in the host for years without clinical manifestations referred to as latent tuberculosis infection. The estimated lifetime risk of TB reactivation is 5–10% and is mostly seen in immunocompromised individuals especially in case of human immunodeficiency virus (HIV) infected population, with a risk of TB approximately 18-fold higher. Disruption of immune control leads to bacterial proliferation and progress to active TB [2]. The rise in multidrug resistant (MDR) and extensively drug resistant (XDR) strains with HIV co-infection makes treatment and control of TB challenging.

In recent years, significant attention has focused on how Mtb actively subverts host cellular machinery through “epigenetic reprogramming”. In the context of infection, epigenetic reprogramming refers to the pathogen-driven manipulation of host chromatin dynamics—encompassing DNA methylation, post-translational modifications, non-coding RNA-mediated regulatory pathways, and miRNA-mediated regulation—to dynamically reshape the immune cell transcriptome without altering the underlying host DNA sequence [3]. This targeted chromatin remodeling plays a central role in host–pathogen interactions by allowing the bacteria to dictate the transcriptional profile of critical immune genes [4]. Specifically, Mtb infection significantly alters post-translational modifications, such as acetylation, phosphorylation and methylation, on the exposed N-terminal tails of host nucleosomal histone proteins. In vitro studies reveal that this pathogen-directed structural alteration of the nucleosome mediates the epigenetic dysregulation of various host immune response pathways [5]. Ultimately, the profound gene expression changes induced by this epigenetic reprogramming led to shifts in host metabolism, altered macrophage polarization, impaired apoptosis, and potentially excessive inflammatory responses, thereby establishing a permissive environment for bacterial persistence.

Mtb modulates the host immune system at multiple levels and has developed strategies to effectively evade both innate and adaptive immune responses. Investigating the virulence factors associated with Mtb, especially the secretory proteins and transcriptional regulators, offers valuable insights and novel targets for TB treatment and drug development. Like other bacterial species, Mtb possesses well-regulated secretion systems that are critical for its virulence and pathogenesis, such as Sec and TAT pathways which are conserved among different microorganisms and Type VII secretion system or ESX secretion system found to be specific only to *Mycobacterium* [6]. The ESX type VII secretion systems (ESX-1 to ESX-5) contribute to phagosomal escape, immune modulation, nutrient acquisition, and intracellular survival [7]. Among them, the ESX-1-secreted virulence factors ESAT-6 and CFP-10 play major roles in pathogenicity by promoting phagosomal membrane disruption, modulating macrophage polarization, and suppressing host immune responses [8,9]. CFP-10 also enhances IFN-γ-mediated immune responses, highlighting its potential as a vaccine candidate [10]. Additionally, the ESX-5 system secretes PE/PPE proteins involved in nutrient uptake, cell surface integrity, and adaptation to stress conditions, thereby promoting survival of Mtb within host cells [11,12].

This review comprises a comprehensive analysis of how Mtb secretory proteins function as nucleomodulins to reprogram host epigenetic machinery and modulate immune responses. It integrates current knowledge on secretion systems, nuclear targeting mechanisms, and epigenetic alterations—including histone modifications and DNA methylation—that collectively facilitate immune evasion and intracellular survival. Furthermore, the review highlights the impact of these processes on macrophage function and disease progression, while critically evaluating emerging host-directed therapeutic strategies targeting epigenetic pathways. Finally, it outlines key challenges and future directions required to translate these insights into effective interventions for tuberculosis control.

## 2. Secretion Systems of Mtb and Delivery of Effector Proteins

Like many other bacteria, Mtb also possesses advanced secretion systems for its efficient pathogenesis. Unlike many Gram-negative bacteria that rely upon classical secretion systems including type 1 to type 6 secretion systems, Mtb carries specialized protein export pathways [6]. For example, Mtb possess the classic Sec and Tat pathways to export proteins across the inner membrane [13]. In addition, as mentioned earlier, it also possesses specialized pathways including the SecA2 pathway [6] as well as the ESX system under the Type 7 secretion system [14]. The most important role of these secretory proteins comes during the process of phagocytosis. Mtb, being an intracellular pathogen invades the phagosome once engulfed by phagocytes and the acidic environment within may affect the fate of the bacteria. However, using these sophisticated secretion mechanisms, Mtb could escape from the antibacterial efficiency of the host [15]. Considering the significant role of these proteins in the establishment of tuberculosis, more studies on this is a need. Each ESX system holds different functions for the advantage of the bacteria. In general, the ESX-1 aids bacteria in evading the host immune system, particularly during phagocytosis. The ESX-2 system mainly helps in the transport of exotoxins to the host. The ESX-3 system, through its substrate, EsxH impairs the activity of CD4+ T cells. ESX-4 influences the cytoskeletal remodeling whereas ESX-5 interferes in the nutrient uptake and immunomodulation [16]. ESX-1 system was the first identified type 7 secretion system in the Mtb family. EsxA and EsxB are the two major secreted proteins from the system. They were formally known as ESAT-6 and CFP-10 [17] where the former is a 6 kDa early secreted antigenic target and the latter is a 10 kDa culture filtrate protein [18]. Unlike the canonical pathways, the ESX system does not possess any signal peptide. Rather, the two major features of them are that they code for conserved Trp-X-Gly (WXG) motif and transmembrane ATPases [19]. More than two decades ago, F. Tekaia and team identified the existence of five ESX clusters, named ESX-1 to ESX-5 [20] among which ESX-4 is the ancestral one [21]. ESX-1 is encoded by the region of difference 1 or RD1. This is completely absent in all the vaccine strains of *M. bovis* and only present in the virulent strains of *M. bovis* and Mtb [22]. As mentioned earlier, EsxA and EsxB being released as heterodimeric proteins could also act as pore-forming toxins [23]. These toxins could form pores in the membrane and aid in the virulence of the bacteria. This system also helps Mtb to invade into the cytosol of the host [24]. The cyclic GMP-AMP synthase recognizes the mycobacterial DNA subsequently activating downstream signaling pathways resulting in the release of IFN-β [25]. With the same function of recognizing the mycobacterial DNA, AIM-2 inflammasome also activates the secretion of IL1β which could act against the pathogen. However, the ESX-1 system could help the pathogen to act against this host immune mechanism [26]. ESX-5 is the most widely studied system after ESX-1. Pro-Glu (PE) and Pro-Pro-Glu (PPE) are the main motifs secreted through this system, which is particularly present in slow growers of the *Mycobacterium* family [27]. Louis S. Ates and colleagues have thoroughly investigated this system in *Mycobacterium*. They have demonstrated its potential role in the capsular integrity. Their study proved that *esx-5* and *ppe10* mutations could result in impaired phagosome rupture which reveals its potential [28]. Huixian Gan et al. demonstrated a phenomenal feature of this system. Generally, in cells that undergo apoptosis, plasminogen activator inhibitor type 2 guards’ annexin-1 from protease attack. As a virulence mechanism, Mtb downregulates the production of this inhibitor. In parallel, the ESX-5 system could result in the production of a protease that could possibly cleave the annexin thereby halting the formation of apoptotic bodies. Interestingly, in such cases the Mtb could transform the apoptosis of the host into necrosis thereby exploiting the immune system [29]. It has been observed that this system favors cell death pathways where it could spread the infection.

Another specialized secretory machinery of Mtb is the SecA2 system. The SecA2 along with the SecY2 protein called SecA2/Y2 is a conserved machinery in *Mycobacterium*. This system supports the transport of large, glycosylated proteins [30]. The SecA1 is ultimately the major system for protein export whereas the SecA2 is a unique system for the secretion of specific proteins [31]. The mycobacterial secA2 pathway is responsible for exporting multiple substrates, hence carries the name multisubstrate secA2 [32]. Even though the SecA2 is present in other bacteria as well, in Mtb its major role is in virulence. Miriam Braunstein and team are a strong group focused on the SecA2 pathway of Mtb, their research has demonstrated more survival in mice infected with secA2 mutant Mtb strain [33]. As mentioned in the ESX system, the SecA2 system also interferes the phagosome but in maturation [34]. The SecA2 system also has the potential to impair the apoptosis of macrophages, thereby providing survival advantage to the pathogen. SecA2 mutant strains of Mtb upon infection in the host have shown reduced levels of pro-inflammatory cytokines. The altered cytokine expression also reveals the immunomodulatory effect of this pathway [13]. Even though numerous studies are moving in this context, still we do not have a complete profile of the potential of these pathways. More molecular unraveling of these export pathways and effectors would bring strong candidates as therapeutic targets. Unique secretory pathways of Mtb are included in Table 1.

## 3. Mycobacterial Secretory Proteins as Nucleomodulins: Targeting Host Histone Modifications

Nucleomodulins represent a specialized class of bacterial effector proteins that target the host cell nucleus and manipulate nuclear regulatory processes to promote pathogen survival and persistence [35]. Unlike conventional virulence factors that function predominantly in the cytoplasm or at host membranes, nucleomodulins released by Mtb exert their effects within the nuclear compartment, thereby influencing fundamental host cellular processes such as transcriptional regulation, chromatin remodeling, and epigenetic modification. Increasing evidence indicates that these effectors alter host chromatin accessibility, histone modification states, and DNA methylation patterns, ultimately reshaping transcriptional networks that regulate immune responses, inflammation, and cellular metabolism [36].

Structurally, nucleomodulins typically exhibit modular architectures that enable multifunctionality within host cells. A defining feature of many nucleomodulins is the presence of nuclear localization signals (NLSs), that consist of short clusters of basic amino acids, primarily lysine and arginine, that are recognized by nuclear import receptors. These signals facilitate interaction with importin proteins, forming transport complexes capable of traversing the nuclear pore complex. Nuclear entry is driven by the Ran-GTP–dependent transport cycle, enabling bacterial proteins to access nuclear chromatin and transcriptional machinery following secretion from the bacterial cell [37]. In addition to localization sequences, they frequently contain enzymatic domains capable of modifying host macromolecules. These catalytic regions may include methyltransferase domains that catalyze histone or DNA methylation, acetyltransferase domains that regulate histone acetylation, phosphatase domains that alter signaling cascades, or protease domains that cleave host regulatory proteins [38]. They could also possess structural motifs that enable direct interaction with host DNA or histone proteins, thereby facilitating chromatin targeting. The modular composition of nucleomodulins allows integration of enzymatic activity with subcellular targeting, enabling precise manipulation of host transcriptional and epigenetic states [35].

### 3.1. Nuclear Targeting and Epigenetic Manipulation by Mtb Secretory Proteins

Mtb has evolved highly specialized strategies to manipulate host cellular processes to establish persistent infection. Among these strategies, direct modulation of host epigenetic mechanisms has gained recognition as a critical determinant of immune evasion and intracellular survival [39]. Epigenetic regulation governs chromatin accessibility and transcriptional responsiveness, allowing pathogens to exert long-lasting control over host gene expression without altering genomic sequences. Emerging evidence suggests that a subset of Mtb secretory proteins may access the host nucleus and modulate chromatin-associated regulatory pathways. However, the extent of experimental evidence supporting direct nuclear localization, chromatin binding, and epigenetic activity varies considerably among individual effector proteins. While direct nuclear localization and chromatin-modifying activity have been experimentally demonstrated for proteins such as Rv1988 and Rv2966c, evidence for similar functions in several other Mtb effectors remains indirect or inferential.

Some secretory proteins have also been implicated in modulation of nuclear chromatin and host gene regulation while others have been reported to localize the nucleus or influence chromatin-associated regulatory pathways. Among the most well-characterized examples is Rv1988, a secreted methyltransferase that has been reported to localize to the nucleus in infected macrophages. Once localized within the nucleus, Rv1988 associates with histone H3 and catalyzes methylation at histone H3 arginine 42 (H3R42), a non-canonical histone modification site located within the nucleosomal core region. This chromatin-targeted methylation suppresses transcription of innate immune response genes, supporting a mechanistic association between bacterial nuclear localization, chromatin remodeling, and transcriptional repression [40]. Another well-characterized example is Rv2966c which has been shown to translocate into the nucleus of infected macrophages, where it binds host genomic DNA and catalyzes methylation at non-CpG cytosine residues. These findings support a role for Rv2966c in host epigenetic modulation, although the broader consequences of its nuclear localization and chromatin interactions remain incompletely defined. Unlike classical CpG methylation events, non-CpG methylation introduces atypical regulatory marks that influence the transcriptional accessibility and stability of chromatin structures. In addition to DNA methylation, Rv2966c has been implicated in regulation of inflammasome-associated pathways, including modulation of NLRP3 activation and downstream pro-inflammatory cytokine production, further supporting its role as a multifunctional nucleomodulin coordinating chromatin modification with immune signaling regulation [41].

Members of the PE/PPE protein families also contribute to host transcriptional regulation. Rv0256c (PPE2) has been reported to localize to the host nucleus and modulate expression of immune-related genes, including inducible nitric oxide synthase (iNOS). PPE2 has additionally been proposed to associate with promoter regions of immune regulatory genes, contributing to transcriptional repression of iNOS expression and reduced nitric oxide production [42]. Importantly, Mtb effectors likely regulate host epigenetic states through both direct chromatin interactions and indirect modulation of host signaling pathways that converge on chromatin-remodeling machinery [43]. Genome-wide methylation studies have revealed widespread epigenetic changes affecting genes involved in cytokine production, apoptosis, and metabolic regulation which contribute to long-term transcriptional reprogramming that supports bacterial persistence and chronic infection [44]. However, for several proposed Mtb histone-modifying effectors, the precise mechanisms governing chromatin targeting and enzymatic activity remain incompletely characterized.

### 3.2. Histone Acetylation and Transcriptional Reprogramming

Beyond promoter targeting and DNA methylation, modulation of host histone acetylation represents a major mechanism through which Mtb nucleomodulins regulate chromatin accessibility. Histone acetylation represents a reversible epigenetic modification controlled by histone acetyltransferases (HATs) and histone deacetylases (HDACs), which regulate transcriptional activity by altering chromatin structure. During Mtb infection, disruption of the HAT–HDAC balance leads to altered acetylation states at promoters of immune-related genes, frequently resulting in transcriptional repression.

Rv3423.1 has been implicated in histone acetylation dynamics through its reported histone acetyltransferase-like activity targeting histone H3 lysine residues such as H3K9 and H3K14. Although its precise chromatin-binding mechanism remains under investigation, some studies suggest that Rv3423.1 may influence anti-inflammatory gene expression patterns, thereby promoting bacterial survival through transcriptional reprogramming. However, direct evidence for nuclear localization and chromatin binding remains limited. In parallel with direct acetyltransferase activity, Mtb infection has been associated with increased recruitment of host HDAC complexes to promoters of immune-responsive genes, resulting in histone hypoacetylation at loci controlling cytokine production, antigen presentation, and antimicrobial responses [45]. ESAT-6, secreted by Mtb, inhibits the expression of class II transactivator (CIITA), a master regulator of major histocompatibility complex class II expression [23]. Although ESAT-6 has not been conclusively demonstrated to function as a direct chromatin-binding nucleomodulin, it can indirectly influence transcriptional programs through modulation of signaling pathways linked to chromatin regulation. Reduced CIITA expression has been linked to altered histone acetylation at promoter regions controlling antigen presentation genes, thereby limiting activation of CD4+ T cells and weakening adaptive immune responses [46]. Similarly, ESX-5–secreted proteins are also associated with alterations in host transcriptional and chromatin-related pathways. Emerging transcriptomic and chromatin accessibility studies suggest possible links between ESX-5 secreted proteins and altered histone modification patterns across immune gene loci, potentially contributing to transcriptional suppression of host defense pathways [47].

### 3.3. Consequences for Immune Gene Expression and Host Defense

**Figure 1:**
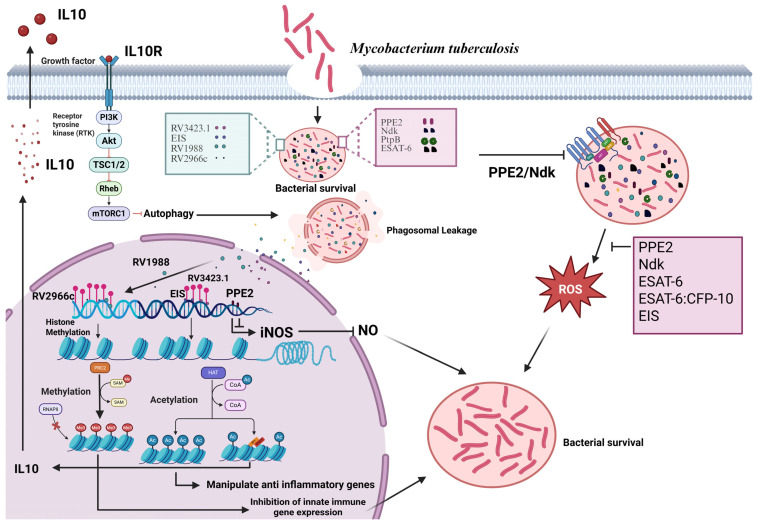
Mechanisms employed by Mtb proteins to manipulate host immune signaling, epigenetic regulation, autophagy, and oxidative responses for intracellular survival: Mtb activates IL-10/IL10R-mediated signaling pathways involving PI3K, Akt, TSC1/2, Rheb, and mTORC1, resulting in suppression of autophagy and enhanced bacterial persistence. Mtb virulence factors including PPE2, Ndk, PtpB, ESAT-6, EIS, RV1988, RV2966c, and RV3423.1 interfere with host antimicrobial mechanisms by inducing phagosomal leakage, inhibiting reactive oxygen species (ROS) production, and reducing inducible nitric oxide synthase (iNOS)-mediated nitric oxide (NO) generation. Simultaneously, bacterial proteins manipulate host epigenetic machinery through histone methylation, DNA methylation, and histone acetylation alterations, thereby modulating inflammatory gene transcription and suppressing innate immune responses. Collectively, these coordinated immune evasion mechanisms establish a favorable intracellular niche that promotes long-term survival of Mtb within macrophages.

The epigenetic modifications induced by Mtb nucleomodulins have profound consequences for host immune gene expression. Many of the targeted genes encode proteins essential for antimicrobial defense, including cytokines, chemokines, and enzymes involved in reactive oxygen and nitrogen species production. Repression of nitric oxide synthase and NADPH oxidase genes reduces production of reactive antimicrobial molecules, thereby impairing macrophage-mediated bacterial clearance. Similarly, suppression of antigen presentation pathways limits activation of adaptive immune responses, allowing bacteria to evade immune detection. Reduced transcription of pro-inflammatory cytokines disrupts signaling pathways required for effective immune activation. Collectively, these coordinated transcriptional changes create a permissive intracellular environment that supports bacterial survival contributing to the chronic nature of tuberculosis infection. Collectively, available evidence suggest that selected Mtb secretory proteins can modulate host epigenetic pathways either directly or indirectly, contributing to transcriptional reprogramming that favors bacterial persistence of host cells. However, the degree of experimental validation differs substantially among proposed nucleomodulins, and additional studies are required to define the precise mechanisms governing nuclear targeting, chromatin interaction, and epigenetic regulation. Through coordinated modulation of histone acetylation, histone methylation, and DNA methylation pathways, these proteins may contribute to the establishment of transcriptionally repressive chromatin environments that suppress antimicrobial responses and promote intracellular survival. The convergence of multiple epigenetic pathways underscores the evolutionary refinement of mycobacterial virulence strategies and highlights the central role of chromatin remodeling in host–pathogen interactions. Understanding these mechanisms provides critical insights into the pathogenesis of tuberculosis and reveals promising opportunities for the development of host-directed epigenetic therapies aimed at restoring protective immune responses [6,48]. Nucleomodulins and other proteins as epigenetic modifiers are depicted and enlisted in Table 2 and Figure 1.

## 4. Epigenetic Reprogramming of Macrophages During Mtb Infection

Macrophages forms an integral part of the innate immune system and are derived from the myeloid progenitor in the bone marrow. They act as primary phagocytic cells that help to clear the cellular debris and apoptotic bodies thereby maintaining immune homeostasis. They serve as the principle host cells for the intracellular survival and persistence of Mtb, and they significantly influence the host immune response to infection [49]. In response to Mtb infection, alveolar macrophages activate a nuclear factor erythroid 2 related factor 2 (NRF 2) mediated transcriptional response characterized by reduced antimicrobial activity and metabolic reprograming that facilitates the supply of iron and fatty acids to Mtb. Evidence suggests that following infection, the Mtb-infected alveolar macrophages migrate to the lung parenchyma from the alveolar lumen and transfer bacteria to other myeloid cells including dendritic cells that carry antigens to lymph nodes and initiate T cell response. This dissemination influences the initiation of adaptive immune response and the susceptibility to TB [50].

Macrophages exhibit different polarized states largely driven by epigenetic mechanisms that enable flexibility between functional programs. This polarization is induced by the exposure of cells to various external factors such as microbial signals, host cytokines, and other environmental stimuli that modify the interaction between transcription factors, DNA, and downstream signaling pathways. Among the histone modification marks, H3K4me has been reported to be strongly associated with macrophage responsiveness to Mtb infection and increased pro inflammatory cytokine production with the enhanced accumulation of H3Kme3 in the promoter regions of tumor necrosis factor (TNF), IL-6, IL-18 etc [51]. Changes in chromatin accessibility can influence the genomic localization and binding of transcription factors such as NF kB and STAT to the DNA. These structural changes can be further remodeled in response to new stimuli allowing macrophages to respond to changing stimuli. Mtb enhances the expression of host sirtuin 2 (SIRT2), NAD+ dependent histone deacetylase, that modifies NFkB-p65 resulting in altered differentiation of macrophage and T cells, favoring bacterial survival [52]. Hypermethylation of NF kB in macrophages during infection with Mtb suppresses the production of various cytokines and chemokines which influence the immune response and bacterial clearance [53]. The processing and presentation of antigen following phagocytosis by the antigen presenting cells to class I and class II MHC molecules are essential for the recognition of invading pathogens by T cells to initiate adaptive immune response. As mentioned in the previous sections, Mtb employs complex strategies to prevent the process of antigen presentation, especially through chromatin remodeling and histone deacetylation, as described in the case of a 19-kDa lipoprotein secreted by Mtb, which inhibits CIITA, the master control factor of MHC class II gene expression by deacetylating H3 and H4 at CIITA promoter in a TLR-2-dependent manner [54]. Itaconate, a byproduct of TCA cycle, competes with both succinate dehydrogenase and α-ketoglutarate that interferes with the activity of DNA dioxygenase TET, leading to DNA hypermethylation and downregulation of inflammatory gene transcription [49].

During the early phase of infection, activation of the innate immune system triggers the production of pro inflammatory cytokines such as interleukin-1β (IL-1β), IL-6, IL-12 and TNF-α in M1 macrophages. This phase is also marked by enhanced uptake of glucose through the upregulation of GLUT 1 and increased glycolysis to generate ATP and metabolic intermediates in M1 macrophages with higher NAD+ consumption while the oxidative metabolism is reduced. However, as the infection progresses, the metabolic state of the macrophage changes to enhanced oxidative phosphorylation and TCA cycle with reduced glycolysis that indicate a shift from M1 to M2 phenotype [55]. Mtb can also alter the metabolism of macrophages during infection to induce M2 polarization by upregulating miR-21 expression that targets phosphofructokinase muscle type (PFK-M) thereby reducing glycolysis. The resulting metabolic alterations reduce the α-KG/succinate ratio of the TCA cycle affecting α-KG dependent demethylases including TET and KDM6B leading to repressive epigenetic modifications including hypermethylation of IL-12B promoter and reduced bactericidal activity [52]. The phagocytosis process and the production of pro inflammatory cytokines and reactive oxygen species are highly dependent on glycolysis whereas the anti-inflammatory response exhibited by M2 macrophage rely more on oxidative phosphorylation and lipid metabolism [56]. During pathogen infection, the M1 macrophage shows significant production of NO from the conversion of arginine into citrulline by NOS/iNOS, primarily induced by IFN-γ. NO thus produced can then be further processed to form reactive nitrogen species (RNS), both exhibiting antimicrobial properties. In contrast, IL-4 or IL-13 stimulate the expression of arginase (ARG 1) that metabolize arginine to ornithine and urea and the further metabolism of ornithine to polyamines and glutamic γ-semialdehyde enhance proliferation of pathogens within M 2 macrophages [57]. The shift between M1 and M 2 phenotypes is an essential requirement for the regulation of immune response against pathogens. Beyond the potential of Mtb in immune evasion and disease progression, they also contribute to cellular transformation and tumorigenesis. The proinflammatory signaling induced during the infection can enhance the NF kB mediated anti-apoptotic signaling and expression of cell cycle regulators, stimulating the proliferation and survival of cells. Similarly, the excessive production of reactive oxygen and nitrogen species induced oxidative stress damage to DNA in macrophages increases mutation rate although they are essential for bacterial clearance [58].

Mtb possess several strategies to escape from the process of autophagy and phagosome formation following the engulfment by macrophages through the utilization of proteins such as protein kinase G (Pkn G), secreted acid phosphatase M (Sap M), protein tyrosine phosphatase A (Ptp A) etc. Protein kinase G mediates the inhibition of phagosome-lysosome fusion and the maturation of autophagosome through the phosphorylation of essential host derived proteins such as those involved in intracellular vesicle trafficking, specifically targeting Rab GTPase, particularly RAB 7, thereby promoting the survival and replication of Mtb within the host macrophage [59]. Pkn G is also reported to interact with another protein called RAB 14, a small GTPase, that plays crucial role in the maturation of phagosome and autophagosome by blocking the hydrolysis of RAB 14-GTP. Phosphorylation of TBC1D4/AS160 (TBC1 domain family member 4) by Pkn G reduces its GAP activity towards RAB 14 in a kinase-dependent manner. This makes Pkn G an efficient bacterial effector protein that modulates host autophagy flux that benefits the survival of Mtb in non-acidified autophagosomes in macrophages [60]. Investigating the regulatory role and the mechanisms of PknG on autophagy along with the identification of Pkn G-interacting host proteins associated with Mtb infection can help uncover new drug targets for TB therapy. The PE/PPE/PGRS family proteins such as PE_PGRS47 and PE_PGRS20 are known to interact with host GTPase Rab 1A which prevents the recruitment of autophagy related proteins thereby blocking autophagy and promoting the survival of bacteria within macrophages [61]. The secreted protein of Mtb, Eis (enhanced intracellular survival), inhibits autophagy by reducing reactive oxygen species (ROS) generation by NADPH oxidase and mitochondria and enhancing the expression of IL 10 via the acetylation of histone H3 and stimulating the Akt/mTOR/p70S6K pathways [62]. Mtb genome encoded PKnG kinase impairs autophagy-lysosome fusion in host macrophages with the inhibition of lysosome acidification by targeting V-ATPase and destabilizing lysosomal membrane integrity. This manipulates the autophagic flux that helps bacteria evade lysosomal degradation, promoting intracellular survival within macrophages [63]. Epigenetic modifications induced by Mtb to evade host immune responses are listed in Table 3.

## 5. Therapeutic Targeting of Mtb-Induced Epigenetic Modifications

The modifications induced by the epigenetic alterations during Mtb infection in a host result in the silencing of antimicrobial activities like autophagy, phagosome maturation, antimicrobial peptide production (LL-37), altered macrophage cell death pathways (↑ LXA4, ↓ TNF), which makes them potential host targets in therapeutics [70,71,72]. Key targets for these therapies also include histone deacetylases (HDACs, sirtuins), DNA methyltransferases (DNMTs), lysine demethylases (KDMs) [73,74]. Unlike conventional medicines like antibiotics that increase antimicrobial resistance by targeting the bacterial pathways which cause mutations and create MDR/XDR strains, targeting these epigenetic factors altered by Mtb reduces the emergence of these strains [53,75]. Unlike mutations, these epigenetic modifications to the hosts are reversible making them very good candidate targets for therapeutics [53].

Host-directed epigenetic therapies have gained popularity over the years due to the increase in emergence of MDR/XDR strains. Instead of focusing on Mtb directly, what they do is focus on the factors that help the bacteria to survive in the host, like these epigenetic modifications [72]. As these epigenetic modifications are reversible, they automatically become a central area of interest as a potential therapeutic target [53].

Histone-modifying enzymes have become one of the most extensively studied epigenetic regulators, particularly histone deacetylases (HDACs). It has been demonstrated that during Mtb infection in macrophages, HDAC1 is upregulated, leading to reduced expression of immune response genes such as *IL-12B*, which is crucial for Th1 immune responses [76]. Studies have also shown that inhibition of HDAC activity reduces bacterial burden and restores the expression of antimicrobial peptides such as LL-37 and β-defensins. A widely accepted in vivo model for HDAC inhibition involves *M. marinum*-infected zebrafish embryos, which showed a significant reduction in mycobacterial load following HDAC inhibition [73]. These findings suggest that epigenetic drugs can indirectly enhance innate immune defense.

Some studies further suggest that inhibition of ZBTB25, which forms a complex with HDAC1, enhances intracellular killing of Mtb through activation of autophagy in macrophages [77]. Trichostatin A (TSA) and suberoylanilide hydroxamic acid (SAHA), two broad spectrum HDAC inhibitors (HDACis), suppress the generation of reactive oxygen species (ROS) and autophagy by downregulating genes associated with autophagy such as *CACNA2D3*. Inhibition studies targeting EGFR (epidermal growth factor receptor) and BRD4 (bromodomain-containing protein 4), a key epigenetic regulator, in in vivo mouse models of tuberculosis demonstrated enhanced lipophagy and restoration of normal angiogenesis, thereby restricting Mtb burden [78].

Another HDAC inhibitor, phenylbutyrate (PBA), enhances host antimicrobial responses by increasing vitamin D-dependent expression of genes including *CAMP* and *CXCL10*, thereby promoting cathelicidin-induced killing of Mtb. PBA also regulates inflammatory pathways to prevent excessive tissue damage. In humans, treatment with PBA combined with vitamin D showed a 28.8% higher culture conversion rate compared to the placebo group [79].

Methyltransferases (MTases) are a class of enzymes which helps catalyze the transfer of methyl group from S-adenosyl methionine (SAM) to substrates like nucleic acids, proteins and other biological macromolecules. There are about 121 identified methyltransferases in the Mtb proteome that use different substrates like DNA, RNA, protein and intermediates of mycolic acid biosynthesis and other fatty acids which are involved in the cellular maintenance within the host [80]. During TB infections, increased activity of DNA methyltransferase (DNMTs) leading to hypermethylation and silencing of immune related genes contributing to immune evasion [74]. Researchers have also found out that when knocking out HsdM (Rv2756c), which is a DNA methyltransferase bacterium, a decrease in susceptibility to isoniazid was seen. Mtb RNA methyltransferase Rv3366 is a SAM-dependent MTase in Mtb which transfers methyl groups from SAM to RNA and causes tRNA/rRNA modifications. Levodopa and droxidopa were found to be potential inhibitors that bind stably with the catalytic site of Rv3366, which was checked through Molecular Dynamics (MD) simulations [81]. Therapeutics targeting these MTases may ultimately reactivate suppressed immune pathways, but more studies have to be carried out to better understand this area.

Some recent research has shown that some of the Mtb protein itself can cause epigenetic manipulations in the host. Studies have reported that Mtb protein Rv2067c can directly modify its hosts histones by targeting histone H3 lysine (H3K79) methylation causing the reduced expression of pro-inflammatory genes and chromatin regulation reducing the host immune response, these proteins also shift the cell death pathways to necrotic pathways helping Mtb in systemic dissemination [82]. Mtb virulent protein Rv3033 inhibits intrinsic apoptotic pathways in macrophages, which helps in bacterial persistence [83]. From a therapeutic perspective, targeting these bacterial proteins like Rv2067c and Rv3033 helps prevent the pathogen from reprogramming the host proteins thereby protecting the host immune function.

Although epigenetic-based therapies show a lot of promise, they are not as effective as conventional anti-tubercular therapy (ATT). The real potential lies in being used as adjuncts with existing drugs. Drugs such as isoniazid and rifampicin which are commonly used as the first line drugs against TB are generally effective but need prolonged administration and are often associated with toxicity. Therefore, if we could combine these two strategies it may be beneficial [84]. There is experimental evidence already supporting this combinatorial therapy, using HDAC inhibitors alongside rifampicin; this also increases the sensitivity of MDR strains to rifampicin [73].

**Figure 2:**
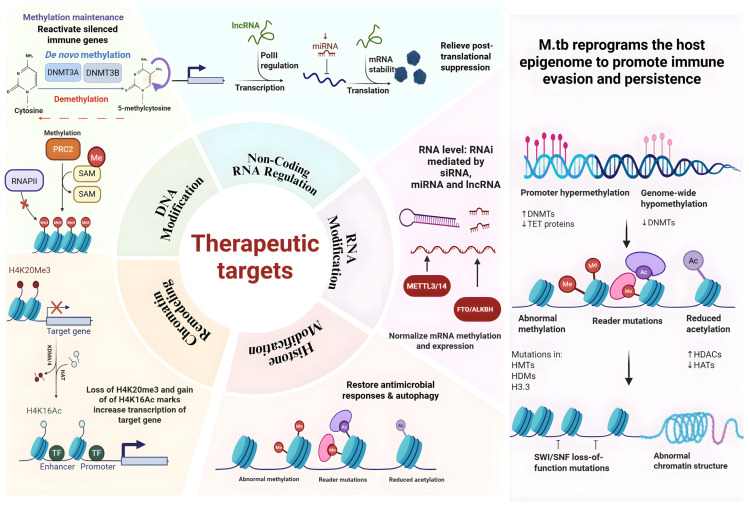
Epigenetic and epitranscriptomic mechanisms exploited by Mtb to modulate host immune responses, promote intracellular persistence and their therapeutic implications. Mtb alters host DNA methylation, histone modifications, chromatin remodeling, non-coding RNA regulation, and RNA methylation pathways to suppress immune gene expression, impair antimicrobial responses, and facilitate immune evasion. Key therapeutic targets including DNMTs, PRC2, METTL3/14, FTO/ALKBH, histone modifiers, and chromatin remodeling complexes are highlighted as potential strategies to restore host defense mechanisms and autophagy during infection.

The other important aspect of epigenetic therapies is to modulate immune response of the host and reduce tissue damage. TB is caused not only by bacterial replication but also through increased inflammatory and immune mediated damage. A summary of host-directed approaches is shown in Table 4 and Figure 2.

## 6. Challenges and Future Perspectives

Despite significant advances in understanding host–pathogen interactions in Mtb, the functional characterization of secretory proteins remains a major mechanistic bottleneck. Much of the current knowledge is derived from heterologous expression systems such as *Mycobacterium smegmatis* or *Escherichia coli*, and from reductionist in vitro biochemical assays. While these approaches have been instrumental for preliminary screening, they fail to recapitulate the highly specialized ESX (Type VII) secretion systems required for native effector export and host interaction [85]. This limitation is particularly relevant for proteins belonging to the PE/PPE families, which require specific mycobacterial chaperone systems for proper folding and secretion [47]. Moreover, many Mtb secretory proteins are enriched in intrinsically disordered regions (IDRs), enabling structural plasticity and context-dependent functionality that are only realized upon interaction with host macromolecules such as chromatin or chaperone networks [86]. Consequently, in vitro validation outside the host cellular environment often produces artifactual or incomplete interpretations.

The complexity of functional validation is further compounded by the extensive redundancy within the Mtb secretome and the dynamic nature of host–pathogen interactions. Large paralogous protein families can compensate for genetic perturbations, masking phenotypic outcomes in conventional knockout models. Addressing this challenge requires multiplexed genetic approaches such as CRISPR interference (CRISPRi) which enable simultaneous repression of gene clusters and reveal non-redundant functions during infections [87]. In parallel, many secretory proteins interact with host targets through transient “hit-and-run” mechanisms that are not readily captured by conventional biochemical assays [88]. Emerging proximity-labelling technologies, including TurboID and APEX2, provide powerful tools to capture these interactions in situ and map dynamic host–pathogen interactomes [89]. Importantly, functional validation must also consider the spatiotemporal dynamics of infection, as effector secretion is tightly regulated by environmental cues such as hypoxia and oxidative stress within host macrophages [90]. Furthermore, the widespread use of immortalized macrophage cell lines introduces experimental bias due to altered baseline epigenetic states, underscoring the need for primary human macrophages and organoid-based systems [91,92].

Another major limitation is the reliance on surrogate and attenuated mycobacterial strains. While *M. smegmatis* and BCG provide experimental convenience, they lack key virulence-associated secretion systems and fail to replicate the full spectrum of host epigenetic reprogramming observed during infection. In contrast, virulent Mtb strains actively manipulate host chromatin through secreted effectors that reprogram immune responses [93,94]. Importantly, even the commonly used H37Rv strain does not fully represent the diversity of circulating the clinical isolates. Hypervirulent strains, particularly those of the Beijing lineage, exhibit enhanced immunomodulatory capacity and distinct epigenetic signatures that promote immune evasion and disease progression [95]. These findings highlight the necessity of incorporating phylogenetically diverse clinical isolates ATAC-seq and CHIP-seq in primary human infection models will be essential to capture strain-specific host modulation [96].

Translational limitations of current tuberculosis models further hinder progress. Conventional murine models lack the genetic diversity of human populations, limiting their ability to capture variability in host epigenetic responses [97]. Additionally, widely used experimental systems fail to incorporate key comorbid conditions such as diabetes and HIV, which profoundly reshape the host epigenome prior to infection [98,99]. Another emerging challenge is the spatial heterogeneity of tuberculosis lesions. Granulomas within the same host can exhibit microenvironments, ranging from hypoxic necrotic cores to resolving fibrotic regions. Bulk epigenomic analyses obscure this heterogeneity, necessitating the integration of spatial transcriptomics and spatial epigenomics to resolve host–pathogen interactions in situ [100,101].

The discovery of novel Mtb secretory effectors with epigenetic functions remain an important frontier. Traditional sequence-based approaches are insufficient for identifying such effectors with host proteins. Instead, many bacterial proteins employ structural mimicry or intelligence-driven structural prediction, particularly AlphaFold, have enabled proteome-wide identification of candidate effectors based on structural similarity [102]. When combined with integrative multi-omics approaches such as dual RNA sequencing and single-cell chromatin accessibility profiling, these tools provide a powerful framework to link bacterial gene expression with host epigenetic remodeling [38]. Genome-wide CRISPRi screening further enables systematic identification of bacterial factors that modulate host chromatin while proximity-labelling strategies such as TurboID facilitate identification of direct host targets [103].

In parallel, host-directed therapies (HDTs) have emerged as a promising strategy to counteract Mtb-mediated immune dysregulation and enhance host antimicrobial responses. Epigenetic HDTs, particularly inhibitors targeting histone deacetylases (HDACs) and DNA methyltransferases (DNMTs), have been shown to restore antimicrobial gene expression, promote autophagic activity in infected macrophages, and enhance intracellular bacterial clearance in preclinical tuberculosis models [104]. However, the immunomodulatory consequences of epigenetic therapies are highly cell-type specific and remain completely characterized. In macrophages, epigenetic modulation primarily affects pathways associated with autophagy, cytokine production, metabolic polarization, and reactive oxygen or nitrogen species generation [105], whereas in dendritic cells, similar interventions may alter antigen processing and MHC-mediated antigen presentation required for effective CD4+ T-cell priming and adaptive immune activation. Furthermore, persistent chromatin remodeling within T lymphocytes may influence T-cell differentiation, exhaustion, and inflammatory responsiveness during chronic infection, thereby contributing to heterogenous therapeutic outcomes [106]. Another major challenge is the potential toxicity and off-target effects associated with systemic epigenetic modulation. Since HDACs, DNMTs, and histone methyltransferases regulate diverse physiological pathways beyond tuberculosis-associated immune responses, prolonged or non-selective inhibition may induce widespread transcriptional dysregulation, hepatotoxicity, hematopoietic abnormalities, or unintended immune suppression in non-infected tissues. Importantly, patients with comorbid conditions such as diabetes often exhibit altered baseline epigenetic and metabolic states, which may further influence therapeutic responsiveness, inflammatory outcomes, and drug-associated toxicity [98,99]. These limitations underscore the need for precision medicine approaches guided by transcriptomic and epigenomic biomarkers, as well as the development of selective epigenetic modulators and targeted delivery systems, including nanoparticle-based therapeutics, to minimize systemic adverse effects while preserving protective immune functions [107,108]. However, the clinical translation of these approaches is complicated by heterogeneity in host immune responses, which necessitates precision medicine strategies guided by transcriptomic and epigenomic biomarkers [109,110]. Moreover, systemic administration of epigenetic modifiers carries the risk of off-target effects, highlighting the need for targeted delivery systems such as nanoparticle-based approaches [107].

Finally, integrating epigenetic insights into vaccine development represents a transformative opportunity. The concept of trained immunity has demonstrated that innate immune cells can undergo long-term functional reprogramming through stable epigenetic modifications [111,112]. These mechanisms contribute to the protective effects of BCG vaccination, but can be actively counteracted by virulent Mtb strains [113]. Future vaccine strategies should therefore incorporate epigenetic considerations, including the rational design of strains lacking immunosuppressive effectors and the use of epigenetic adjuvants to enhance immune priming [108,114,115]. Additional targeting of hematopoietic stem cells to induce long-term immune reprogramming offers a promising avenue for durable protection [116,117]. Collectively, integrating advances in epigenetics, systems biology, and precision medicine will be critical for overcoming current limitations and achieving effective tuberculosis control.

## Figures and Tables

**Table 1:** Summary of major unique secretory pathway in Mtb.

Secretion System	Key Substrates	Functions	References
ESX-1	ESAT-6	Membrane pore formation, phagosomal rupture, cytosolic access and interferon release	[24]
	CFP-10		[25]
ESX-5	PE	Maintains capsule integrity, immune modulation	[28]
	PPE		
SecA2	SodA	Oxidative stress resistance, catalase peroxidase activity, serine-threonine kinase activity	[13]
	KatG		
	PknG		

**Table 2:** Mtb secretory proteins acting as nucleomodulins targeting host epigenetic modifications.

Protein	Type of Enzymatic Activity	Host Target	Epigenetic Modification	Mechanism of Action	Functional Outcome in Host	Reference
Rv1988	Histone methyltransferase	Histone H3	Methylation of H3R42	Binds histone H3 and catalyzes non-canonical methylation at nucleosomal core region	Suppression of innate immune genes (NOX1, NOX4, NOS2)	[1]
Rv2966c	DNA methyltransferase	Host genomic DNA	Non-CpG cytosine methylation	Binds host DNA and methylates cytosine residues outside CpG islands	Alters transcription factor binding and regulates inflammatory genes	[2,3]
Rv3423.1	Histone acetyltransferase-like protein	Histone H3	Acetylation at H3K9/H3K14	Modifies chromatin-associated histone lysine residues	Promotes transcription of anti-inflammatory genes	[4]
Rv0256c (PPE2)	DNA-binding nucleomodulin	iNOS promoter	Transcriptional repression	Binds promoter region of iNOS gene	Decreased nitric oxide production and antimicrobial response	[5,6]
EIS	Acetyltransferase	Histone H3	Histone H3 acetylation	Increases histone acetylation and induces IL-10 expression	Suppresses inflammatory responses and autophagy	[7,8]
ESAT-6	ESX-1 secretory effector	CIITA- associated chromatin	Indirect histone acetylation modulation	Alters transcriptional regulation of antigen presentation genes	Reduced MHC-II expression and impaired T-cell activation	[9,10]

**Table 3:** Epigenetic modifications induced by Mtb to escape host immune defence mechanisms.

Immune Evasion Mechanism	Epigenetic Modification	Target/Mechanism	Reference
Autophagy inhibition	Histone methylation	Phosphoribosyltransferase of Mtb inhibits autophagy in an mTOR-dependent manner by the hypermethylation of H3 lysine 9 and lysine 27 at the promoter of Atg 5 and Atg 7 genes inhibition.	[64]
Macrophage polarization	Histone methylation	Increased H3K4me3-induced expression of AKT and ARG2 promotes M2 polarization	[65]
Antigen presentation	Histone phosphorylation and acetylation	Suppression of MHC II expression on Mtb infected macrophages by CCR5-mediated histone phosphorylation and acetylation.	[66]
Apoptosis	Histone methylation	H4K20 monomethylation by histone methyl transferase 8, SET 8, affects apoptosis by enhancing M2 polarization.	[67]
Cytokine production	DNA methylation	Hypermethylation of host NF kB and suppression of cytokine production	[68]
	RNA modification	Inhibition of TLR2/MyD88 signaling by miR-27b	[69]

**Table 4:** Host-directed epigenetic therapies in tuberculosis.

Category	Epigenetic Modifiers	Mechanism	Examples	Reference
Histone Modification Inhibitors	HDACs (including non-selective)	Modulates host immunity by suppression of either autophagy, Ros generation or enhancing Vitamin D-dependent antimicrobial killing while regulating inflammation	Trichostatin A(TSA), suberoylanilide hydroxamic acid (SAHA), phenylbutyrate.	[78,79,81]
Methyltransferase (MTase) inhibitors	Methyltransferases (DNA, RNA)	Disrupts pathogenic methylation by inhibiting host DNMTs to reactive silenced immune genes and blocking bacterial Rv3366 to prevent essential RNA modifications	Levodopa, droxidopa	[81]

## Data Availability

The original contributions presented in this study are included in the article. Further inquiries can be directed to the corresponding author.
